# Feasibility and Outcomes of *Meta Salud Diabetes* Behavioral Health Intervention: A Pilot Study of a Community Health Worker-Administered Educational Intervention to Prevent Cardiovascular Disease and Its Complications among Hispanic Patients with Type-2 Diabetes

**DOI:** 10.3390/ijerph20216968

**Published:** 2023-10-24

**Authors:** Tomas Nuño, Maria Rocio Torres, Sheila Soto, Refugio Sepulveda, Benjamin Aceves, Cecilia Ballesteros Rosales

**Affiliations:** 1 Department of Epidemiology and Biostatistics, Mel and Enid Zuckerman College of Public Health, University of Arizona, Tucson, AZ 85721, USA; 2 Rural Health Office, University of Arizona, Tucson, AZ 85721, USA; torresm1@arizona.edu; 3 Department of Public Health Practice and Translational Research, Mel and Enid Zuckerman College of Public Health, University of Arizona, Phoenix, AZ 85004, USA; ssoto2@arizona.edu (S.S.); refugio@arizona.edu (R.S.); crosales@arizona.edu (C.B.R.); 4 Division of Health Promotion and Behavioral Science, San Diego State University, San Diego, CA 92182, USA; baceves@sdsu.edu

**Keywords:** type-2 diabetes, cardiovascular disease, disparities, behavioral intervention, hispanic

## Abstract

Background: Hispanics in the United States experience a greater burden of type-2 diabetes (T2D), with a prevalence rate (17%) more than twice that of non-Hispanic whites (8%). Cardiovascular disease (CVD) is the leading cause of death among people with T2D. A culturally appropriate behavioral health intervention that addresses healthy lifestyle promotion is an impactful approach for health systems with scarce medical resources and a high prevalence of chronic conditions, including obesity and high blood pressure, which increase the likelihood of CVD mortality among type-2 diabetics. Purpose: To assess the feasibility and outcomes of a behavioral intervention to decrease CVD and complications in a Hispanic diabetic population. Methods: *Meta Salud Diabetes* (MSD), a behavioral intervention effective in a Mexican population, consists of a 13-week intervention addressing CVD and T2D knowledge and risk reduction. It was implemented in a sample of Hispanic diabetic patients from two federally qualified health centers (FQHCs). Clinical and behavioral variables were measured at baseline, postintervention, and 1-year follow-up. Results: The feasibility of MSD was rated as successful by all FQHC staff and well-received by both staff and study participants, with positive remarks about the culturally relevant components of the intervention. The sample size was *n* = 30 (baseline), *n* = 23 (postintervention), and *n* = 19 (1-year follow-up). Of note, quantitative results showed trending decreases in Hba1c (7.06; 6.80; 6.30), blood pressure (132/83; 126/80; 123/78), and total cholesterol (160; 159; 154). Conclusion: MSD is a feasible intervention and can address the need to improve health outcomes among Hispanic patients with T2D.

## 1. Introduction

Cardiovascular disease (CVD) is one of the leading causes of mortality among Hispanics in the United States (US) [1]. Hispanics diagnosed with type-2 diabetes (T2D) are at greater risk of complications from CVD and have an increased risk of morbidity and early death [1,2]. Overall, Hispanics have the highest prevalence of age- and sex-adjusted total diabetes compared to non-Hispanic whites and non-Hispanic blacks [3,4]. T2D has increasingly become a leading cause of early death and morbidity in Hispanic populations, both in the US and internationally [5]. In Arizona, approximately 590,916 people, or 10.7% of the adult population, have been diagnosed with diabetes [6]. Studies have reported that Mexican American adults have a higher prevalence rate of T2D compared to other Hispanic subgroups and to non-Hispanic Whites [7].

These disparities are linked to a host of environmental, biological, and social factors. For example, Hispanic adults are more prone to developing T2D as a result of genetic predispositions and social determinants that prevent them from accessing adequate health care and disease management [8]. Hispanics may lack access to information on healthy choices, including eating habits and maintaining an active lifestyle, which immediately places them in a risky and challenging position for T2D prevention and CVD complications among Hispanics with T2D [8].

Culturally tailored, evidence-based educational and lifestyle interventions that target modifiable factors among Hispanics are needed to reduce chronic disease health disparities on a population level. Culturally tailored lifestyle interventions for T2D prevention appear to be modestly effective in reducing the risk for T2D among Hispanics in the US [9]. For CVD, there is some evidence that culturally appropriate interventions provide potentially efficacious strategies for cardiovascular risk improvement among Hispanics [10]. Potential effective strategies include the use of *promotoras* (also known as community health workers), bilingual materials/classes, and appropriate cultural diet and exercise modifications. *Promotoras* create a liaison between underserved and medically vulnerable populations, such as Hispanics, and health providers by facilitating access to health services and education. Additionally, the World Health Organization (WHO) endorses peer support interventions for T2D management, an approach that promotes the management of modifiable risk factors for both T2D and comorbid conditions, including CVD [11].

*Meta Salud Diabetes* (MSD) is a behavioral intervention that was found to be effective in a Mexican diabetic population [12]. MSD is the result of collaborations between binational researchers with years of experience developing interventions to address chronic disease prevention in the US and Mexico, with a focus on the US–Mexico border. Previous interventions that are the basis for MSD showed that the use of *promotoras* and the prevention of chronic diseases was achievable [13,14]. MSD is the most up-to-date enhancement of these previous intervention efforts, with a focus on preventing CVD complications in a population diagnosed with T2D. MSD was tested in a parallel, two-arm, cluster-randomized controlled trial (RCT) to evaluate the effectiveness of MSD for CVD prevention among Mexicans with T2Ds, compared with usual care, where the unit of randomization was the health center. A slightly adapted US version of this intervention (to account for units of English vs. metric measurements) was conducted in two Southern Arizona federally qualified health centers (FQHCs). The aim of this pilot study was to assess the feasibility and clinical and behavioral outcomes of the MSD intervention in a different clinical and community setting with a culturally similar population. The Spanish-speaking Hispanic population of Southern Arizona has much in common with the Mexican population of Sonora, Mexico. Information on the clinical and behavioral risk factors for CVD among Hispanic adults with T2D was assessed. This pilot study allowed for a comparison of the impact of the MSD intervention in both of these populations that experience CVD and T2D health disparities.

## 2. Materials and Methods

### 2.1. Study Sites

The University of Arizona (UArizona), in collaboration with *El Colegio de Sonora* in Hermosillo, Sonora, Mexico, developed the MSD curriculum to decrease CVD and its complications in a Mexican population diagnosed with T2D. The methodology and results for MSD in Mexico have been previously published, but a brief description of the intervention and curriculum is provided in the methods section below [12,15]. MSD was implemented in two FQHCs in Southern Arizona that serve a large Spanish-speaking population. El Rio Community Health Center (ERCHC) is in an urban community in Pima County in Tucson, AZ and provides medical, dental, and disease prevention services. Tucson is 60 miles away from the US–Mexico border and is the second largest city in Arizona, whose Hispanic population comprises 44.2% of the population [16]. Mariposa Community Health Center (MCHC) is located in Santa Cruz County, a rural/border community that separates both Nogales, Arizona and Nogales, Sonora, Mexico. On average, 94.6% of the population is of Hispanic origin and 77.6% are Spanish-speaking in this area [16]. MCHC is the main health center that offers medical, mental, dental, and health education [14]. Both of these FQHCs are the largest providers in their counties that respond to the high demand of health care needs of low-income, uninsured patients. *Promotoras* are part of their highly skilled team that educates communities on health-related topics such as chronic disease prevention and management.

### 2.2. Participant Eligibility and Recruitment

*Promotoras* recruited participants at the ERCHC and MCHC clinics. The study participants met specific inclusion criteria, including (1) age of at least 18 years or older, (2) have a Hispanic background, (3) speak and understand Spanish, (4) residents of the selected county, (5) be diagnosed with prediabetes or T2D, (6) provide written informed consent, and (7) have the desire to participate and attend the scheduled sessions.

### 2.3. Intervention and Curriculum

The MSD intervention consisted of 2 h class sessions delivered over a 13-week period, providing educational information to encourage sustainable behavioral change to prevent disease complications, including nutrition education, CVD and T2D education, the social environment, and the adoption of physical activity. MSD was delivered in Spanish by an assigned *promotora* from each FQHC. The changes made to the US version of MSD included weights in ounces and pounds and heights in inches and feet. All other topics and the intervention structure remained the same. The 13-week calendar presented topics in the following order: (W1) introduction to MSD; (W2) living a healthy life with T2D; (W3) are you at risk of developing CVD?; (W4) maintaining a healthy weight; (W5) benefits of physical activity; (W6) glucose and sugar; (W7) everything you need to know about high blood pressure, salt, and sodium; (W8) control your cholesterol, eat less fat; (W9) is our community healthy?; (W10) enjoy life with emotional well-being; (W11) effective management of T2D is a shared responsibility; (W12) enjoy healthy meals with friends and family, meal demonstration; and (W13) review/graduation. The weekly 2 h sessions were broken down into the following seven segments: (S1) assessment of personal goal; (S2) review of last week’s topic; (S3) review of weekly goal; (S4) introduction to the week’s topic; (S5) lecture and activities; (S6) physical activity; and (S7) summary of the session.

The MSD intervention was a goal-driven program that introduced four primary goals: (1) increase in physical activity, (2) reduction of sweet-beverage consumption, (3) reduction of high-fat and high-carbohydrate products, and (4) increase in fruit and vegetable consumption. A handbook was given to study participants to facilitate each session by incorporating the weekly goals and objectives, important and useful information pertaining to the topics, a log chart to track their continuous progress regarding their personal goals and clinical outcomes (blood pressure, glucose, and weight), and additional recommendations for an active lifestyle. The MSD curriculum also utilized social support as a key component to keep participants engaged in the class sessions and mutual support to meet individual goals. Group activities like aerobic physical activity, creative skits, in-class tasks, and the discussion of debates cultivated a sense of community and a learning environment. This binationally developed intervention was conducted in a culturally relevant and context-specific setting with Mexican food, music, arts, customs, and entertainment used during demonstrations associated with preventive measures.

### 2.4. Outcomes and Measures

#### 2.4.1. Implementation Science Outcome

For this study, we assessed the feasibility of the intervention. Feasibility is defined as the extent to which a new treatment, or an innovation, can be successfully used or carried out within a given agency or setting [17]. Feasibility is invoked retrospectively as a potential explanation of an intervention’s success or failure, as reflected in poor recruitment, retention, or participation rates [18].

#### 2.4.2. Clinical Outcomes

The clinical outcomes of interest in this study were classified into three categories, (1) biological, (2) behavioral, and (3) psychological, which followed the same measurement protocols and self-administered survey sources from the parent grant. The clinical outcomes included the Framingham Risk Score (FRS), hemoglobin A1c (Hb1c), fasting blood glucose, blood pressure (systolic and diastolic), triglycerides, total cholesterol, high-density lipoprotein cholesterol (HDL), and low-density lipoprotein cholesterol (LDL). The anthropometric measures were body mass index (BMI), weight, waist, hip, and height. The FRS variable expressed as a percentage predicts the risk of developing CVD complications (coronary death, myocardial infarction, coronary insufficiency, angina, ischemic stroke, hemorrhagic stroke, transient ischemic attack, peripheral artery disease, and heart failure) over a 10-year period. In this research, the adapted model was based on the following predictors: age, sex, total cholesterol, HDL cholesterol, BMI systolic blood pressure, smoking and T2D status, and blood-pressure treatment. FRS is valid in the United States, indicating a low risk below 10%, a moderate risk between 10–19%, and a high risk above 20%.

The CVD behavioral risk factors considered moderate physical activity, time sitting, sweet-beverages consumption, and vegetable intake. The psychological indicators for CVD comprised perceptions of overall, physical, and mental health through the Health-Related Quality of Life (HRQoL) score [19].

The Problem Areas in Diabetes Questionnaire (PAID) scale measures the emotional distress that patients may develop after being diagnosed with T2D [20]. This instrument uses a 20-item scale that is commonly employed in intervention studies to identify emotional change over time. Each criterion ranges from 0 points (not a problem) to 4 points (a serious problem). The sum of all the item’s points is multiplied by 1.25, generating the PAID score; a total score of 40 or higher indicates severe distress caused by T2D.

#### 2.4.3. Data Collection and Management

All data were collected at baseline (the week before the class sessions started at each FQHC) and postintervention (3-month and 12-month follow-up assessments) by the research team from UArizona. The study was conducted from April 2018 through October 2019. During this enrollment session, a research member provided informed consent prior to the data collection, per the study protocol. Eligible participants received a hard copy of the consent in Spanish attesting to their understanding of the benefits and potential harms. Sociodemographic information, clinical indicators, and administered self-reported surveys on CVD prevention behaviors and risk factors were gathered. REDCap was used to capture and manage data, which is a clinical and translational research database authorized by the UArizona. The Human Subjects Protection Program at the UArizona approved this study.

#### 2.4.4. Statistical Analyses

The statistical analyses combined all participants from both FQHCs to increase the sample size and power. However, as our project is a pilot study, we present statistical analyses as an exploratory effort to compare results from the MSD cluster RCT conducted in Mexico. Descriptive statistics of the original participants were calculated at baseline. Unadjusted mean differences and proportions were calculated at baseline, 3 months, and 12 months for the total sample. Linear mixed regression models were computed to account for the violation of independent observations assumption on multiple measures from individuals. Variables adjusted for potential confounding included sex, age, socioeconomic status (SES), education, and marital status. Stata 16 was used to perform statistical analyses.

## 3. Results

**Figure 1:**
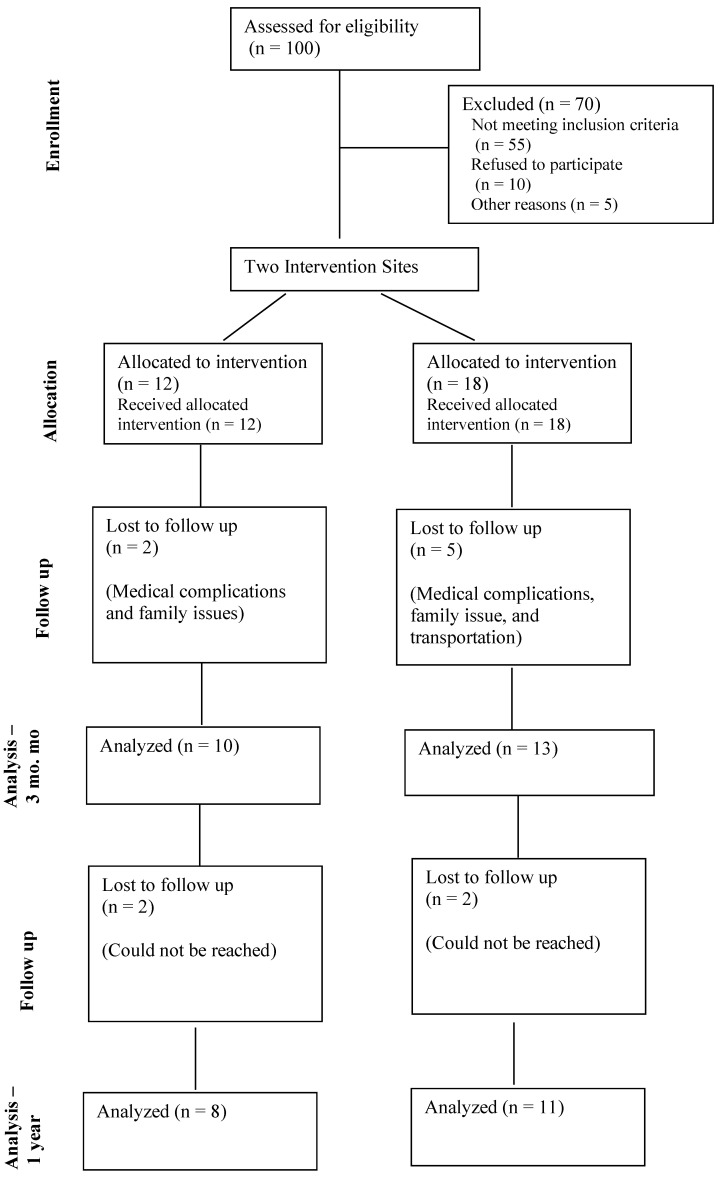
Study Enrollment Flowchart.

To assess feasibility, we evaluated the number of sessions attended by the study participants and feedback from the staff and the study participants. The recruitment rate was 30% (100 subjects were invited to participate, with 30 replying positively). However, the retention rate was higher; 23 out of the 30 participants completed the 13-week MSD intervention (76.6%). At 1-year follow-up, 19 study subjects participated in the data collection and survey assessment (Figure 1). The reception from the participants was positive; they followed instructions each session and were eager to participate in the different dynamics that were assigned. The reception from the two *promotoras* and four staff members of the FQHCs was positive. MSD was well-received by both study staff and study participants, with positive remarks about the culturally relevant components of the intervention. The feedback from all six FQHC staff was that the MSD was successfully carried out in their centers with an interest in continuing to offer MSD to their Hispanic patient population.

Table 1 shows the baseline characteristics of participants from both ERCHC and MCHC. Participants were mostly females (70%); the mean age was 61 years; were married or had a partner (63.3%); their annual income was higher than USD 12,000 (36.7%); were medically diagnosed with T2D (86.7%); were born in another state or country other than Arizona (90%); and did not report an alcohol use (63.3%).

Unadjusted and adjusted means of the clinical and anthropometric outcomes are exhibited in Table 2 The FRS declined by 2.5% from baseline to postintervention (3 months) after adjustment (95% CI: −5.3, 0.1, *p* = 0.06) though nonstatistically significant. The HbA1c level showed a slight decrease from 3 to 12 months by 0.5% (95% CI: −0.1, 0.0, *p* = 0.05). Both of the adjusted means of the average of systolic and diastolic blood pressures were statistically significantly lower at 3 months; systolic pressure fell by 6.7 mmHg (95% CI: −13.1, −0.4, *p* = 0.04) and diastolic pressure by 3.0 mmHg (95% CI: −6.6, 0.6, *p* = 0.1). The level of triglycerides decreased after 12 months by 71.4 mg/dL (95% CI: −123.1, −19.7, *p* = 0.01). 

Table 3 demonstrates the behavioral risk factors regarding sedentary lifestyle and diet. The time seated dropped by 9.4 min per day (95% CI: −86.7, 68.0, *p* = 0.81). On the contrary, the consumption of sweet beverages was statistically significantly lower by 0.5 portions (95% CI: −0.9, −0.2, *p* = 0.002). For the psychological outcomes, the PAID questionnaire score showed a statistically significant decrease in emotional distress by 12 points (95% CI: −23.4, −2.0, *p* = 0.02). On the other hand, participants reported a lower score on the health-related quality of life (HRQoL) at the end of the program. The score showed a difference of 0.2 (95% CI: −0.3, 0.7, *p* = 0.48).

## 4. Discussion

The MSD intervention study in two Southern Arizona FQHCs was found to be feasible by the staff who administered it. MSD was successfully carried out within these agencies and settings and showed a fairly promising retention rate and positive reception by the participants. For the clinical outcomes, a slight promising effect on HbA1c among Hispanic adults with T2D was also found. The unadjusted and adjusted models demonstrated declines at 12 months, implying a positive effect from the program regardless of the possible role of confounders. The average of HbA1c was above 7 at baseline, declined to 6.8 at 3 months, and was 6.3 at 12 months. We expect that the curriculum components, such as physical activity and nutrition, made a difference in this clinical outcome and were associated with CVD complications. On the other hand, blood glucose had a baseline of 158.1 mg/dL, which increased to 159.2 at 3 months and decreased to 145.1 at 12 months. Even though the former result was not statistically significant, it showed a decreasing trend that could have crossed the cutoff point of the “controlled T2D” measure (140 mg/dL) if the sample size were larger.

The similarity of results between the MSD intervention results in Mexico and the results of our study in Arizona is striking. Despite differences in study design and sample size, our results comparing pre- and postintervention mirror the cluster-RCT results. Both studies show decreases in FRS, HbA1c level, systolic blood pressure, and PAID distress score at postintervention. This provides evidence of the robustness of the MSD intervention to affect positive change in different clinical and community settings. It could also imply that the MSD intervention is well-tailored for the Spanish-speaking Hispanic, Mexican-origin population of the US as well as Mexico. Culturally tailored lifestyle interventions for T2D and CVD prevention appear to show modest to moderate effectiveness in reducing risk for T2D and CVD among Hispanics in the US, and it has been seen in US–Mexico border settings [9,10,21,22,23,24]. An innovative aspect of MSD was combining information and resources for the prevention of both CVD and T2D. For those with T2D, the content of MSD can lead to behavioral changes that lead to the prevention of CVD complications. Though the study population of interest was those diagnosed with T2D, the information on MSD was also relevant and appropriate for those diagnosed with prediabetes. Previous research has shown that a joint prevention/self-management intervention led by CHWs for low-income Hispanics with T2D and with prediabetes is feasible and cost-effective [25]. MSD incorporated effective strategies to impact a Hispanic population, including the use of *promotoras,* Spanish-language materials/classes, and appropriate cultural diet and exercise activities.

To address the escalating CVD and T2D epidemic among the Hispanic population of the US, evidence-based, cost-effective, and scalable interventions are needed. MSD can fill these gaps on several levels. The intervention was first implemented in Mexico in a large cluster RCT to establish the evidence of its effectiveness. The implementation of MSD in two FQHCs in Arizona showed that the intervention could be feasible in different community and clinical settings, with minor changes in measurement units for the US Hispanic population. The important issues of culturally appropriate materials and implementation are key factors addressed by the MSD intervention. MSD represents an opportunity to promote evidence-based practice flow that can work not only from a high-income country to low- and middle-income countries, but vice versa. Our future plans include the implementation of MSD as a larger prospective cohort study in five FQHCs in Southern and Central Arizona, to have a greater reach to the US Hispanic populations in the state. Additionally, we plan to partner with collaborators in Southern California for the implementation of MSD in the US–Mexico border settings there. Our expectation is that MSD will be feasible in these settings and that a larger sample size will allow for statistical comparisons that are significant and mirror the RCT conducted in Mexico. Given the social nature of the intervention and the need to be implemented in person, the timeline to start our study post-COVID is in place.

### Limitation and Strengths

Our study did have several limitations. The small sample size of our pilot study was a limiting factor in our statistical analyses, which affected the power to reject the null hypothesis. Given that our study was a pilot study, the statistical analyses should be considered as exploratory. However, it was noteworthy that we still found statistically significant results and significant differences in measures of effect. Also, our intervention-only study design is not as robust as the cluster RCT of the MSD Mexico study. Another limitation was the use of the FRS, which has been demonstrated to overestimate CVD risk in US Hispanic populations, though no alternative or more accurate measures of CVD risk among Hispanics have been identified [20,26]. Another limitation is loss-to-follow-up and, in particular, the missing data in the 1-year follow-up assessment. Our study data would have been much stronger if we had complete data that followed the trends shown throughout the study period. Lastly, our study was conducted pre-COVID-19 and does not take into account changes in social environment situations post-COVID-19. With the cluster RCT results from the Mexico study being published in 2021, our study is a natural progression to show the impact that MSD can have in the US as well as Mexico. Our study had several strengths as well. The history of the MSD intervention development from evidence-based interventions and long binational collaboration ensured MSD would be developed in a culturally appropriate manner. Also, conducting this pilot of MSD in FQHCs was a strength and allowed for MSD to be taught by *promotoras* and to reach the Hispanic population of interest. Future research should include partnering with FQHCs to administer MSD as a robust multilevel intervention method that can have a greater impact on implementation science and clinical outcomes.

## 5. Conclusions

MSD is a feasible intervention and can potentially address the need to improve health outcomes among Hispanic patients with T2D. Comprehensive public-health interventions and policies are needed to lower CVD risk among those who have T2D and to prevent the development of T2D among prediabetics, which could lower the future burden of CVD and T2D among the Hispanic population of the US.

## Figures and Tables

**Table 1:** Baseline characteristics of original participants at baseline (N = 30).

Participant Characteristics	All (N = 30)
Age (years), mean (SD)	61.2 (8.2)
Gender, n (%)	
Female	21 (70.0)
Males	9 (30.0)
Education (years), mean (SD)	11.6 (4.4)
Birth Place, n (%)	
Arizona	3 (10.0)
Other state/country	27 (90.0)
Civil Status, n (%)	
Married or partnered	19 (63.3)
Nonmarried/partnered	11 (36.7)
Annual Income, n (%)	
≤12,000	11 (36.7)
12,001–20,000	7 (23.3)
20,001–50,000	7 (23.3)
≥60,001	2 (7.4)
Diabetes Status, n (%)	
Diabetes	26 (86.7)
Prediabetes	4 (13.3)
Previously Diagnosed with CVD, n (%)	
Yes	8 (26.7)
No	22 (73.3)
Medication for diabetes as prescribed, n (%)	
Yes	28 (93.3)
No	2 (6.7)
Insured, n (%)	
Yes	29 (96.7)
No	1 (3.3)
Current Drinking, n (%)	
None	19 (63.3)
1–4 times/week	3 (10.0)
1–3 times/month	4 (13.3)
1–11 times/year	4 (13.3)

**Table 2:** Clinical and anthropometric outcomes at baseline (N = 30) and 3-month follow-up (N = 23).

Variable	Time	Unadjusted Mean (SD)	*p*-Value ^a^	Adjusted ^b^ Mean (SD)	*p*-Value
CVD Risk	Baseline	20.3(12.1)			
	3 months	18.7 (12.8)			
	Difference (95% CI)	−1.8 (−4.4, 0.8)	0.17	−2.5 (−5.3, 0.1)	0.06
HbA1c, (%)	Baseline	7.06(0.3)			
	3 months	6.8(0.3)		-	
	Difference ^a^ (95% CI)	−0.3 (−0.6, 0.1)	0.17	−0.4 (−0.8, 0.1)	0.09
	12 months	6.3(0.3)			
	Difference ^b^ (95% CI)	−0.5 (−0.9, −0.1)	0.02	−0.5 (−0.1, −0.0)	0.05
Blood Glucose, (mg/dL)	Baseline	158.1(10.6)		-	
	3 months	159.2(12.3)		-	
	Difference ^a^ (95% CI)	1.1 (−23.7 25.9)	0.93	4.9 (−22.9, 32.7)	0.73
	12 months	145.1(13.2)			
	Difference ^b^ (95% CI)	−14.0 (−41.9, 13.8)	0.32	−18.9 (−50.4, 12.7)	0.24
Average Systolic Pressure ^c^, (mmHg)	Baseline	132.1(2.5)		-	
	3 months	126.0(2.9)		-	
	Difference ^a^ (95% CI)	−6.1 (−12.0, −0.22)	0.04	−6.7 (−13.1, −0.4)	0.04
	12 months	122.6(3.2)			
	Difference ^b^ (95% CI)	−3.4 (−10.1, 3.3)	0.32	−1.8 (−9.1, 5.4)	0.62
Average Diastolic Pressure ^c^ (mmHg)	Baseline	82.9(1.5)		-	
	3 months	79.6(1.7)		-	
	Difference (95% CI)	−3.3 (−6.4, −0.1)	0.04	−3.0 (−6.6, 0.6)	0.1
	12 months	77.6(1.9)			
	Difference ^b^ (95% CI)	−2.0 (−5.5, 1.6)	0.28	−1.8 (−5.9, 2.4)	0.4
Total Cholesterol, (mg/dL)	Baseline	159.7(6.2)		-	
	3 months	158.7(6.8)		-	
	Difference ^a^ (95% CI)	−1.0 (−11.6, 9.6)	0.85	−3.9 (−15.1, 7.3)	0.5
	12 months	153.9(7.2)			
	Difference ^b^ (95% CI)	−4.7 (−16.5, 7.0)	0.43	−8.1 (−20.7, 4.5)	0.21
HDL Cholesterol, (mg/dL)	Baseline	44.3(1.9)			
	3 months	44.3(2.1)			
	Difference (95% CI)	−0.06 (−3.2, 3.4)	0.97	−0.4 (−3.9, 3.1)	0.81
	12 months	44.1(2.2)			
	Difference ^b^ (95% CI)	−0.2 (−3.6, 3.3)	0.92	−0.04 (−3.9, 3.9)	0.99
LDL Cholesterol, (mg/dL)	Baseline	71.8(6.0)			
	3 months	71.3(6.5)			
	Difference (95% CI)	−0.5 (−9.7, 8.7)	0.91	−1.5 (−12.2, 9.2)	0.79
	12 months	73.7(6.8)			
	Difference (95% CI)	2.4 (−8.1, 12.9)	0.66	0.2 (−12.4, 12.7)	0.98
Triglycerides, (mg/dL)	Baseline	214.6(18.4)			
	3 months	241.4(21.2)			
	Difference (95% CI)	26.7 (−15.0, 68.5)	0.21	18.8 (−27.0, 64.6)	0.42
	12 months	184.2(22.8)			
	Difference (95% CI)	−57.1 (−104.0, −10.3)	0.02	−71.4 (−123.1, −19.7)	0.01
Anthropometrics					
BMI, (kg/m^2^)	Baseline	32.1(1.0)			
	3 months	32.8(1.1)			
	Difference (95% CI)	0.7 (−0.9, 2.3)	0.39	0.8 (−1.0, 2.7)	0.36
	12 months	32.7(1.1)			
	Difference (95% CI)	−0.1 (−1.9, 1.6)	0.88	−0.1 (−2.2, 1.9)	0.9
Average Weight ^c^, (lbs)	Baseline	189.1(7.3)			
	3 months	194.1(7.7)			
	Difference (95% CI)	5.0 (−4.0 13.9)	0.28	4.5 (−5.9, 14.8)	0.4
	12 months	193.4(7.9)			
	Difference (95% CI)	−0.6 (−10.5, 9.2)	0.90	−0.4 (−12.0, 11.2)	0.95
Average Waist ^c^, (in)	Baseline	43.9(1.0)			
	3 months	43.5(1.0)			
	Difference (95% CI)	−0.4 (−1.8, 1.0)	0.55	−0.1 (−1.5, 1.3)	0.88
	12 months	43.8(1.0)			
	Difference (95% CI)	0.3 (−1.1, 1.7)	0.66	0.3 (−1.2, 1.9)	0.66

^a^ Mixed regression models, ^b^ Adjusted for sex, age, SES, education, marital status, ^c^ Average of three measurements.

**Table 3:** Behavioral factors at baseline (n = 30) and 3-month follow-up (n = 23).

Variable	Time	Unadjusted Mean (SD)	*p*-Value ^a^	Adjusted ^b^ Mean (SD)	*p*-Value
Physical Activity					
Moderate Physical Activity (min/week)	Baseline	492.2(112.0)			
	3 months	525.7(133.8)			
	Difference (95% CI)	33.5 (−228.6, 295.8)	0.80	39.5 (−260.0, 339.0)	0.80
Time Seated (min/day)	Baseline	229(30.0)			
	3 months	194.9(36.0)			
	Difference (95% CI)	−34.1 (−104.3, 36.1)	0.34	−9.4 (−86.7, 68.0)	0.81
Diet					
Sweet Beverages (portions in mL)	Baseline	0.9(0.2)			
	3 months	0.1(0.3)			
	Difference (95% CI)	−0.8 (−1.5, −0.1)	0.01	−0.5 (−0.9, −0.2)	0.002
Vegetable Consumption (portions per day)	Baseline	1.7(0.3)			
	3 months	1.7(0.3)			
	Difference (95% CI)	0.01 (−0.6, 0.7)	0.93	−0.2 (−0.9, 0.6)	0.66

^a^ Mixed models, ^b^ Adjusted for sex, age, SES, education, marital status.

## Data Availability

The data presented in this study are available on request from the corresponding author. The data are not publicly available due to privacy.
